# Introduction to Oxidative Stress in Biomedical and Biological Research

**DOI:** 10.3390/biom5021169

**Published:** 2015-06-09

**Authors:** Michael Breitenbach, Peter Eckl

**Affiliations:** Department of Cell Biology, University of Salzburg, Hellbrunnerstrasse 34, 5020 Salzburg, Austria; E-Mail: peter.eckl@sbg.ac.at

**Keywords:** oxygen radicals, ROS, proteomics, mass spectrometry, electron spin resonance spectroscopy, respirometry, redox homeostasis, aging, photosynthesis, adaptation

## Abstract

Oxidative stress is now a well-researched area with thousands of new articles appearing every year. We want to give the reader here an overview of the topics in biomedical and basic oxidative stress research which are covered by the authors of this thematic issue. We also want to give the newcomer a short introduction into some of the basic concepts, definitions and analytical procedures used in this field.

The term “stress” was first used in the biomedical literature as a description of hyperactivity in the hormone system, in particular concerning the corticosteroids of the adrenal cortex [1]. The author nicely summarized some 20 years later [2] how the idea of stress, stress response, and homeostasis as a dynamic equilibrium gradually developed into a highly useful idea in general physiology and the study of diseases. He saw “stress” primarily as a factor causing disease, and even today, as exemplified by this thematic issue, modern stress research is still largely concerned with pathomechanisms of human disease. Today we know that in many of those stress sitations, in fact redox processes play a major role.

The concept of physiological stress in general for a long time was ill-defined in physicochemical terms, it was “fuzzy” like the concept of oxidative stress. It took decades before a clearer picture could be established by delineating the molecular mechanisms of stress generation, stress defense and stress signaling.

The term “oxidative stress” was coined only 30 years ago [3]. In a recent review article [4] the history and development of this scientific concept is vividly described. The concept is based on earlier work by Selye (loc. cit), and inspired by early publications related to oxygen toxicity, often connected with the problem of aging [5,6,7], the metabolism of oxygen (and other) radicals in biological systems [8], the gradual development of our understanding of mitochondrial physiology [9,10], “mitochondrial” aging research [11,12], and the study of redox imbalance in cells and organisms [13]. Redox imbalance according to one definition is another name for oxidative stress which is based on the Nernst equation taking into account all the redox couples present in the cell or in the different cellular subcompartments [14]. Another more practical and operational definition of oxidative stress is given by Lushchak: “Oxidative stress is a situation when steady-state ROS concentration is transiently or chronically enhanced, disturbing cellular metabolism and its regulation and damaging cellular constituents” [15].

The yeast system of molecular genetics is ideally suited to study the relationship between different kinds of stress (heat stress, oxidative stress by different oxidants like H_2_O_2_, paraquat, and diamide, osmotic stress, salt stress, *etc.*) by studying the reaction of the cells to low inducing stress conditions by transcriptomic and proteomic methods. This was beautifully shown by the group of Ian Dawes in a series of papers [16,17,18,19]. The adapted cells after the intial treatment are more resistant to high “killing” stresses than without the hormetic conditioning. These experiments led in part to very surprising results. On the one hand, the adaptive reaction of the yeast cell included not only resistance to the same primary stress that was initially applied, but also to other seemingly unrelated stresses. An example is given by cross-reactivity to conditioning by oxidants and by heat. On the other hand, this cross-reactivity was by no means universal. Also the direct genomic reactions to a primary stress were not universal as shown by the transcriptome. Therefore, the concept of “generalized stress”or a “generalized stress reaction” is certainly an overstatement, if not a mistaken concept altogether, although the idea of a generalized stress response is still to be found in the literature [20]. Also, different oxidants did not overlap in the genomic reaction and the resistance which they induced [16]. There is every reason to believe that a similarly complicated system of cross-reactivity and cross-adaptation also exists in higher cells. However, this was never tested in detail due to the greater technical difficulties in higher cell systems.

Herrmann and Dick have recently summarized the main routes of research in redox biology, which now seem to be of pre-eminent importance [21]. Figure 1 shows a grapical representation of these fields of research.

Presently, an exponentially increasing number of scientific articles and books are appearing which are dealing with oxidative stress in one way or the other. To give an overview and a few examples, we are listing here the numbers of publications, as of May 2015, and give a few typical examples. 

In the last two years alone, 59 books appeared dealing with oxidative stress. The large majority of these books are medical, treating oxidative stress in connection with a large number of different specific diseases (for instance: cancer, neurodegeneration in Alzheimer’s or Parkinson’s disease, arteriosclerosis, *etc.*), sources of nutritional antioxidants for the prevention of disases, pro-oxidant actions of antioxidants, and in anti-aging medicine, and so on. Only a minority deals with other topics such as oxidative stress in plants, or in veterinary medicine. Looking at the titles of these books, it becomes clear that in some cases concepts are desribed for the public which have no firm founding in science. To give the most prominent example, there are books and articles about the anti-aging effects of many antioxidative plant-derived compounds, although clinical trials did generally not show the promised healing or rejuvenation effects [22]. In the last few years, no fewer than five international research journals (*Free Radical Research*, *Free Radicals in Biology and Medicine*, *Redox Reports*, *Antioxidant Redox Signaling*, *Redox Biology*) dealing with oxidative stress were launched, as mentioned by Helmut Sies in his recent review article [4]. Over 1,990,000 hits in Google scholar use the term oxidative stress. Furthermore, there are 627,000 results on lipid peroxidation and 1,840,000 on lipid oxidation, the major downstream targets of oxidative stress. In PubMed, in just under 32,000 articles oxidative stress is named in the title of the papers, of which close to 3500 are review articles.

**Figure 1:**
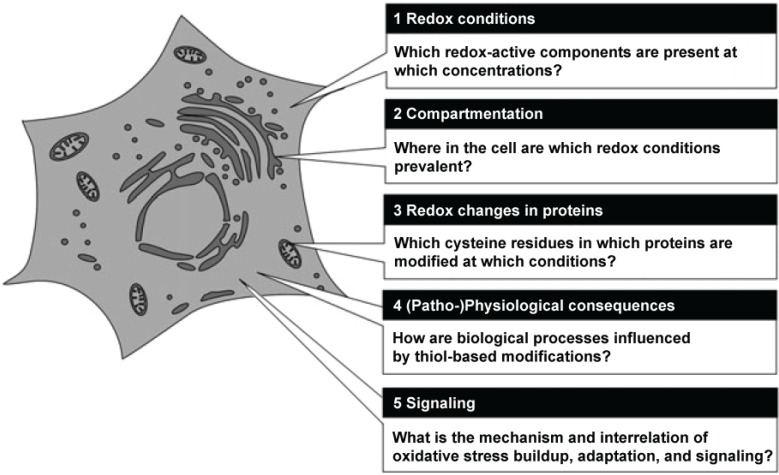
Central questions in redox biology. Source: Herrmann and Dick, *Biological Chemistry* 393, 999–1004 (2012) modified, with permission.

For the present thematic issue, we have assembled an internationally well-recognized team of contributing authors, who are not clinicians, but as biologists work in biomedical science and are presenting here an important and new aspect of oxidative stress research, related either to a specific medical problem, or contributing to the basic understanding of oxidative stress.

Reliability of research in redox biology depends very much on the reliability of the anlytical methods used, and progress in the field very often was enabled by the development of new analytical methods. Two of the chapters in this thematic issue deal with key innovations in methodology that are at present still further improved every year. First of all, modern methods in protein mass spectrometry have made it possible to study the oxidation of individual cysteine residues as shown in the chapter by Verrastro *et al*. The redox cycle of SH groups in proteins can either activate or inactivate catalytic SH groups or fine-tune the catalytic activity. When the SH groups concerned act as a redox sensor they undergo a redox cycle starting with the formation of a sulfenic acid as described also in the chapter by Breitenbach *et al.* Analyzing the oxidation state of those SH groups on specific proteins throughout the “cysteine proteome” [23] using high throughput mass spectrometry methods opens up a huge field of research into the regulatory network of the cell. This is exemplified, for instance in excellent work from the laboratory of Ursula Jakob on the proteomic analysis of the cellular redox interaction network [24].

Another methodological advance concerns respirometry. Being able to measure with high precision the oxygen metabolism of mitochondria in small samples is a key technological advance which is treated by Makrecka-Kuka *et al.* Oxidative stress in a number of diseases and in aging can profoundly change such mitochondrial parameters as the P/O ratio or the respiratory control ratio (RCR). Such measurements are required with high precision and, in a clinical setting, with high speed. They can help to define such highly dangerous disease states as ischemia reperfusion injury in stroke and heart infarction, and even more in septic shock, as shown in the chapter by Weidinger and Kozlov.

Being able to measure superoxide, the primary product of oxygen toxicity, with high sensitivity and selectivity is a key requirement of redox biology. This is possible through the development of reliable chemiluminescence and electron spin resonance methods, as described elsewhere [14,25].

**Figure 2:**
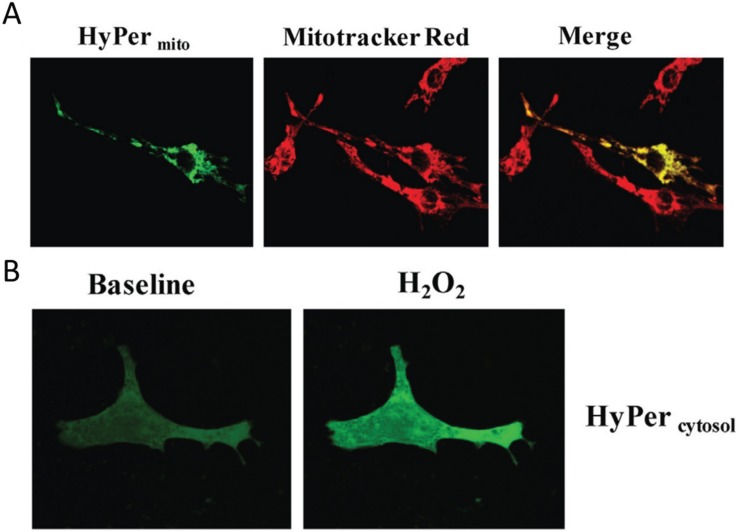
Visualization of H_2_O_2_ in subcellular compartments. (**A**) Neonatal rat ventricular cardiomyocytes transfected with the genetically encoded mitochondria targeted H_2_O_2_ sensor HyPer*_mito_* Mitochondrial localization is confirmed by colocalization with the mitochondrial dye Mitotracker Red. Cells were analyzed after monoamine oxidase ativation with dopamine; (**B**) Neonatal cardiomyocytes expressing cytosol-targeted HyPer*_cytosol_* before and after addition of 100 µM H_2_O_2_. Source: Kaludercic *et al.*, *Frontiers in Physiology*, 5, article 285 (2014) with permission.

Another and very important series of methodological advances in the last few years are redox sensitive fluorescent tags or dyes that enable to study the localization and quantification of redox active compounds, and in fact the redox potential in different subcellular compartments *in vivo* [21,26,27,28]. These studies make it very clear that redox-active micro-domains exist in the known subcellular compartments, such as mitochondria and the ER, and even in the cytoplasm of cells. A whole series of protein reporters based on the bacterial H_2_O_2_ sensitive HyPer protein domain has been developed (reviewed in [26]). An alternative method uses roGFP and related constructs to measure the redox potential in subcellular domains [29,30]. Figure 2 shows an example of the H_2_O_2_ detected by mitochondrial HyPer in heart muscle cells metabolically stimulated by dopamine.

The fluorescence strictly co-localizes with the mitochondria-specific Mitotracker dye, while in a control experiment cytoplasmic H_2_O_2_ is detected with a different HyPer construct which localizes strictly to the cytoplasm of the cell. In summary, these relatively recent results clearly show the compartmentation of redox-active compounds and signals *in vivo* in eukaryotic cells.

Duvigneau *et al.* in their chapter present a bioanalytical method to measure bilirubin, an important antioxidative molecule in liver cell systems, which is relevant in studying heme degradation by heme oxygenase taking place in the liver under oxidative stress.

Oxidative stress in aging in general and in aging human skin in particular is treated here by Rinnerthaler *et al.* It is now nearly universally accepted that oxidative stress is not only associated with but also plays a major role in the aging processes of all cells [31].

The metabolism of heme and of iron in the cell is essential for survival of every cell, because iron, like copper, is a redox-active transition metal needed for the most basic reactions of cellular metabolism. The transition between ferric and ferrous iron ions, just like the one between cupric and cuprous copper ions is within the range of the biological redox potential of the cell and is important for the reactions catalyzed. On the other hand, the ferrous state in iron and the cuprous state in copper can take part in the Haber-Weiss and Fenton reactions thereby creating OH radicals, the most dangerous reactive oxygen species known. Therefore, in living cells, these metal ions are usually bound by chelating ligands which prohibit the dangerous reactions, and their redox transitions and the transport of the ions into the cell are tightly regulated. This is described in detail in the chapter by Bresgen and Eckl. Likewise, heme degradation, which is a physiologically necessary process, is tightly regulated and avoids the production of free iron ions. The physiological problems related to this process and its importance for liver diseases is presented by Duvigneau *et al.*

Oxidative stress as a consequence of increased activity of muscle and of other tissues is a topic of high relevance to sports medicine and to the study of exercise in hypoxia. Steinbacher and Eckl discuss the effects of oxidative stress in muscle training including the effect of ROS and ROS derived lipid peroxidation products as signaling substances to increase the production of defense enzymes and antioxidants and the effects on gene expression in muscle that lead to adaptation and beneficial health effects of training.

The influence of oxidative stress on the ion flux activity of calcium-activated potassium channels in nerve and muscle are reviewed by Hermann *et al.* This constitutes an important link of oxidative stress with the activity of the central nervous system.

Netzer *et al.* discuss the effects of hypoxia on the metabolism of fat cells. Hypoxia can under certain circumstances induce oxidative stress, and it can exert a positive influence on fat cell metabolism to counteract insulin resistance in type 2 diabetes. Recently, the hypothesis was presented by J. D. Watson that type 2 diabetes might be a “redox disease” [32], however, as far as we know Watson’s suggested clinical trials in this direction are not yet under way.

Diabetes type 2 clearly leads to all the molecular markers of oxidative stress in combination with other disease markers that have more directly to do with the increased level of free glucose in serum and in the cells. Nowotny *et al.* give a comprehensive discussion of advanced glycation end products (AGEs) in type 2 diabetes, treating the synthesis as well as the role in the pathomechanism of these highly complex products of irregular oxidative sugar metabolism.

The influence of oxidative stress and other stress situations on a global shift in the metabolic network of the cell was investigated and is presented here by the group of Ralser (Pedrafita *et al.*). They show that under oxidative stress not only the familiar shift from glycolysis to the pentose phosphate shunt takes place, but also a large number of “unwanted” side reactions which must be accounted for and to some degree repaired to ensure survival of the cell.

Oxidative stress is encountered by cells after bacterial infection and in a state of inflammation [33] and is in fact part of the primary innate immune defense of the body, including also the well known oxidative burst of macrophages and monocytes. Stoiber *et al.* show that oxidative stress in such a situation is also involved in the formation of so-called extracellular DNA traps which can help killing of bacteria.

Oxidative stress plays a very important role in the interaction of the human body with the common parasite *Candida albicans*. This fungal commensal organism can in special cases (immune deficiency) develop into a life-threatening systemic infectious disease. The strategies used by *C. albicans* to evade the oxidative burst of human macrophages and the strategies used by macrophages to mitigate the oxidative stress response (including filamentation) in the fungus are treated in the chapter by Da Silva Dantas *et al.*

The group of Jamieson is presenting an account of anti-inflammatory substances from the fruits of haskap (*Lonicera caerulea*) from Canada, which were tested in a human cell culture assay after pro-inflammatory stimulation with lipopolysaccharide (Rupasinghe *et al.*). 

An important chapter of this thematic issue deals with what one could call second messengers of oxidative stress, namely lipid oxidation and the biological effects of aldehydic lipid oxidation products, among which 4-hydroxynonenal (HNE) appears to be the most important, since it contributes to both physiological and pathophysiological mechanisms. For example, mitochondrial uncoupling is dependent upon HNE, as are other cellular reactions such as proliferation and apoptosis. Schaur and co-authors give a concise overview on its formation, chemical ractions with macromolecules in the cell, and the consequences thereof.

Graham Noctor in his chapter on reactive oxygen and its control in plants is treating the production of oxygen in photosynthesis, the avoidance of deleterious side reactions leading to ROS, and also the use of these reactive oxygen species for special biosynthetic and defense reactions in plants.

To conclude this introductory chapter we are coming back to the still fragmentary studies of molecular mechanisms of both the primary response of a cell to oxidative stress and to the adaptive response that enables a cell to survive after a change of the metabolic makeup, or alternatively, the final mechanism of apoptotic or necrotic programmed cell death. These processes require a signaling cascade that is only in outline known today. However, oxidative signaling is mentioned or treated in part by nearly all chapters of this thematic issue. We will briefly describe the role of peroxiredoxin, NADPH oxidases and H_2_O_2_ signaling to transcription factors without going into detail, as this topic is treated in the chapter by Breitenbach *et al.* There is increasing evidence that in human cells the signal created by plasma membrane receptors for peptide hormones and cytokines, like EGF [34], insulin [35,36] or PDGF [37], is transmitted by H_2_O_2_ to phosphotyrosine phosphatase 1B (PTP1B). In this way, the catalytic cysteine SH group of PTP1B is oxidized and inactivated. This leads to an increase in the phosphorylation state of the target proteins. Peroxiredoxin takes part in this reaction. It is the quantitatively most important detoxifying enzyme for H_2_O_2_ and organic hydroperoxides, but it is also a highly regulated module helping in transmission of the H_2_O_2_ signal. This is achieved by reversible hyperoxidation of the peroxidative SH of peroxiredoxin thus creating a strong but highly localized feed-forward reaction which increases the local H_2_O_2_ concentration needed for signal transmission. Compartmentation of signaling is important to avoid oxidative damage in the cell and is achieved by localization of NADPH oxidase [38] and by localization of H_2_O_2_ [39] which was shown by the HyPer method mentioned above. They showed that internally created H_2_O_2_ acts locally and does not easily diffuse to the whole cytoplasmic space. This signal leads to phosphorylation and nuclear transfer of the relevant transcription factors which are different in yeast cells and human cells [40]. Examples are Yap1 in yeast cells and NF-κB in human cells. Alternatively, nuclear transfer and transcription activity can also be regulated by direct oxidation of the transcription factors. One of the target genes of NF-κB is Nox4, the NADPH oxidase which is functional in this signaling cascade [41]. Obviously, many questions concerning this signaling cascade remain open at present. But we believe that the relationship between oxidative stress defense and oxidative signaling by H_2_O_2_ is one of the most exciting topics in oxidative stress research in years to come.
